# *Helicobacter cinaedi* Hepatic Cyst Infection with Bacteremia

**DOI:** 10.3201/eid2503.180936

**Published:** 2019-03

**Authors:** Tetsuya Suzuki, Satoshi Kutsuna, Motoyuki Tsuboi, Masayuki Ota, Kayoko Hayakawa, Norio Ohmagari

**Affiliations:** National Center for Global Health and Medicine, Tokyo, Japan

**Keywords:** Helicobacter, hepatic cyst, immunocompetence, bacterial infection, bacteremia, bacteria, Helicobacter cinaedi, Japan

## Abstract

*Helicobacter cinaedi* is an enterohepatic bacillus that causes infections of various manifestations. We report a novel case of hepatic cyst infection with bacteremia caused by *H. cinaedi* in an immunocompetent woman in Japan. Further research is warranted to identify the epidemiologic and clinical features of *H. cinaedi* infection.

*Helicobacter cinaedi* is a gram-negative, spiral-shaped enterohepatic bacillus found in the digestive tracts of humans and other animals (*1*). Many reports have described that *H. cinaedi* can cause infections in immunocompromised patients (*2*). In recent decades, however, several cases of immunocompetent patients with *H. cinaedi* infection have been reported (*3*). Although various manifestations of *H. cinaedi* infection have been described, to our knowledge, no cases of hepatic cyst infection have been reported. We report a case illustrating an *H. cinaedi* hepatic cyst infection with bacteremia in an immunocompetent patient in Japan.

In July 2017, a 73-year-old woman was referred to the National Center for Global Health and Medicine (Shinjuku, Japan) because of a 2-day history of abdominal pain, vomiting, and fever. She had schizophrenia and had been hospitalized in a psychiatric hospital for >20 years. At admission, her body temperature was 37.5°C, and other vital signs were stable. Physical examination revealed a slight tenderness on her abdomen with mild rebound tenderness. Her laboratory findings showed elevated leukocytes (10,170 cells/mL, reference range 3,300–8,600 cells/μL), neutrophils (87%, reference 40%–71%), and C-reactive protein (11.7 mg/dL, reference 0–0.14 mg/dL). Total bilirubin (0.8 mg/dL), aspartate aminotransferase (13 U/L), alanine aminotransferase (10 U/L), alkaline phosphatase (264 U/L), and gamma-glutamyl transferase (21 U/L) levels were within reference ranges. Enhanced computed tomography (CT) showed slight ascites, but no apparent signs of bowel obstruction. We found a large hepatic cyst (63 mm in diameter) on the left liver lobe (Figure); the cyst had been 44 mm in diameter a month earlier, when she was hospitalized because of a small intestinal obstruction.

**Figure:**
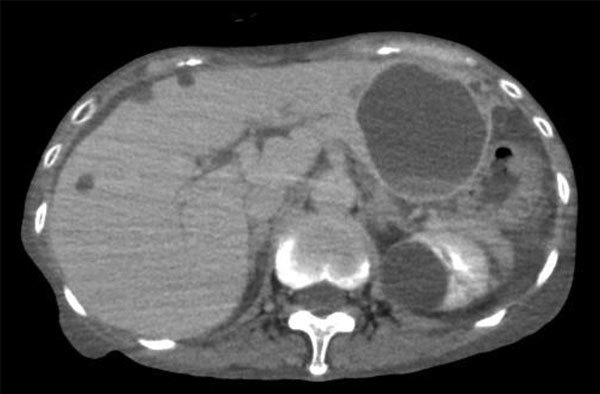
Enhanced computed tomography image showing a large hepatic cyst (63 mm in diameter) on the left liver lobe in a patient with *Helicobacter cinaedi* infection on the day of hospital admission, Tokyo, Japan, July 2017.

On the day of hospital admission, we obtained 2 blood culture samples and started the patient on a regimen of piperacillin/tazobactam. Her fever persisted. On day 5, blood culture results were positive for spiral-shaped gram-negative rod bacteria in both sets of aerobic bottles (after 80.6 h and 104.5 h) (BACTEC, Becton, Dickinson and Company, https://www.bd.com). Enhanced CT on day 5 revealed enlargement of the hepatic cyst from 63 mm to 69 mm in diameter. The patient’s tenderness was most pronounced in the upper abdomen. We suspected hepatic cyst infection and placed an ultrasound-guided drainage tube, by which we obtained ≈100 mL of purulent liquid. The patient’s fever subsided rapidly, within 1 day after drainage. 

We identified the isolates from the blood cultures as *H. cinaedi* by using matrix-assisted laser desorption/ionization-time of flight mass spectrometry (score 2.07). Subsequent PCR testing of pus obtained by drainage also gave a positive result for *H. cinaedi*.

We collected >100 mL of pus in the first 2 drainage days (hospital days 5–6). The patient showed defervescence on day 6. We changed her antibiotic to ampicillin/sulbactam on day 10. A nonenhanced CT scan indicated that the hepatic cyst had collapsed from drainage on day 17. We removed the drainage tube on day 18 and stopped the antibiotic regimen on day 22. The patient’s condition was stable after drainage, and she was discharged to the previous psychiatric hospital on day 24.

We report a novel case of hepatic cyst infection caused by *H. cinaedi*. Uwamino et al. reported a retrospective observational study of community-acquired *H. cinaedi* bacteremia (*3*), showing that cellulitis was the most common manifestation in community-acquired cases, whereas bacteremia without any specific focus was the leading type of infection in healthcare-associated and nosocomial settings. In the case we describe, the patient was in a nosocomial setting at the onset. Thus, primary occult bacteremia is the leading hypothesis. However, *H. cinaedi* is an enterohepatic bacterium, and the patient had undergone surgery for an adhesive small intestinal obstruction 1 month before. Her medical and surgical history might have increased the intraintestinal pressure and induced the hepatic cyst infection through biliary reflux.

The patient had schizophrenia but was not immunocompromised beyond her surgical history. Many cases of *H. cinaedi* infection have been reported in immunocompromised hosts (*2*), but reports of *H. cinaedi* infections in immunocompetent patients have been increasing (*3*). Matsumoto et al. showed that *H. cinaedi* bacteremia was found in only 0.06% of total blood samples (*4*); none of the patients in their study were HIV-positive, but many were immunocompromised by other conditions. Kiehlbauch et al. also conducted a retrospective study of *H. cinaedi* bacteremia and found that 45% of patients were HIV-positive (*5*). *H. cinaedi* infection can occur regardless of a patient’s immunologic or environmental status.

*H. cinaedi* infections are often reported in Japan. Miyake et al. reported that the *H. cinaedi* detection rate has increased after introduction of the BACTEC system (*6*). We also used BACTEC bottles. The widespread use of this type of blood culture bottle throughout Japan might contribute to the positivity rate of *H. cinaedi*. 

We report a case of *H. cinaedi* hepatic cyst infection with bacteremia. *H. cinaedi* infection can occur in both nosocomial and community-acquired situations and in both immunocompromised and immunocompetent patients; its manifestations vary quite widely. Although the positivity rate of *H. cinaedi* is very low, it might still be overlooked. Further research is warranted to identify the epidemiologic and clinical features of *H. cinaedi* infection.
